# Implementation and dissemination of physical activity-related health competence in vocational nursing training: study protocol for a cluster-randomized controlled intervention trial

**DOI:** 10.1186/s13063-024-08153-2

**Published:** 2024-05-15

**Authors:** Johannes Carl, Eva Grüne, Johanna Popp, Verena Hartung, Klaus Pfeifer

**Affiliations:** 1 Department of Sport Science and Sport, Friedrich-Alexander-Universität Erlangen-Nürnberg, Erlangen, Germany; 2 Institute for Physical Activity and Nutrition, Deakin University, Geelong, Australia

**Keywords:** Apprenticeship, Cluster randomized controlled trial, Education, PAHCO, Physical activity, Students, Work ability

## Abstract

**Background:**

Although the nursing sector gains growing importance in an aging society, students representing the future workforce often show insufficient health. Acknowledging the health-enhancing effects of adequate physical activity, the educational system in Bavaria, Germany, has recently integrated the promotion of physical activity-related health competence (PAHCO) into the nursing curriculum. However, it cannot be assumed that PAHCO has sufficiently permeated the educational practices and routines of the nursing schools. Therefore, the goal of the present study is to examine and compare the effectiveness as well as implementation of different intervention approaches to address PAHCO in the Bavarian nursing school system.

**Methods:**

We randomly assign 16 nursing schools (cluster-based) to four study arms (bottom-up, top-down led by teachers, top down led by external physical activity experts, control group). Schools in intervention group 1 (IG-1) develop multicomponent inventions to target PAHCO via cooperative planning (preparation, planning, and implementation phase). Intervention groups 2 and 3 (IG-2, IG-3) receive both an expert-based intervention (developed through intervention mapping) via trained mediators to address PAHCO. External physical activity experts deliver the structured PAHCO intervention in IG-2, while teachers from the nursing schools themselves conduct the PAHCO intervention in IG-3. In line with a hybrid effectiveness implementation trial, we apply questionnaire-based pre-post measurements across all conditions (sample size calculation: *n*_final_ = 636) to examine the effectiveness of the intervention approaches and, simultaneously, draw on questionnaires, interview, and protocol data to examine their implementation. We analyze quantitative effectiveness data via linear models (times-group interaction), and implementation data using descriptive distributions and content analyses.

**Conclusion:**

The study enables evidence-based decisions about the suitability of three intervention approaches to promote competencies for healthy, physically active lifestyles among nursing students. The findings inform dissemination activities to effectively reach all 185 schools of the Bavarian nursing system.

**Trial registration:**

Clinical trials NCT05817396. Registered on April 18, 2023.

**Supplementary Information:**

The online version contains supplementary material available at 10.1186/s13063-024-08153-2.

## Introduction

### Current challenges in nursing care

With a global nursing workforce of 27.9 million, nurses represent the largest occupational group in the health sector and form the backbone of the health care system [1]. However, the shortage of nurses is a major concern in most of the world’s countries, thus creating a global health emergency [2]. This nursing situation is also evident in Germany, with 1.7 million nursing professionals facing five million people in need of care [3, 4]. In the future, this mismatch will further aggravate due to demographic change and the associated decline in young professionals, on the one hand, as well as a rising demand for care services from the aging population, on the other [5]. As a result of this shortage, nurses face increased occupational demands and stress, which can lead to health problems that, in turn, translate into high levels of sick leave and turnover. Corresponding to a vicious cycle, this can further exacerbate nursing shortage [6–8].

The German government has recognized the problem of nursing shortage and is committed to improve the attractiveness of nursing care. With law reforms, such as the (German) Nursing Staff Reinforcement Act (German: “Pflegepersonal-Stärkungsgesetz”), the Act on the Nursing Professions (German: “Pflegeberufereformgesetz”), and the Concerted Action on Nursing scheme (German: “Konzertierte Aktion Pflege”), specific actions are implemented to tangibly improve working and training conditions for nursing staff. In summary, these initiatives specifically aim to attract more nurses (e.g., promoting vocational nursing training, recruiting nursing professionals from abroad), to ensure better payment (e.g., establishing mandatory wage floors), and to implement human resource management, occupational safety, and health promotion (e.g., ensuring staffing levels, strengthening nurses’ health-literate behavior) [9].

### Opportunities for health promotion in vocational nursing training: the role of physical activity-related health competence

Within the scope of these legal reforms described above, vocational nursing training in Germany was reformed to increase the attractiveness and modernity of vocational nursing training and, therefore, to gain more nursing students. In 2020, health politicians replaced the previously separately specialized vocational training programs of pediatric nursing and patient care as well as geriatric nursing with a vocational training program that qualifies general nurses with a broader focus (Act of the Nursing Professions; Ger: Pflegeberufsgesetz, PflBRefG). A total of 52,140 nursing students have commenced the 3-year generalist vocational nursing training in 2022 [10]. Importantly, the vocational nursing training also contains health promotion within its curricula [11] to raise health awareness of nursing students at an early stage and, thus, support future nursing professionals’ work ability. Given the potential of physical activity (PA) to make a significant contribution to individuals’ health [12, 13], promoting PA is an important element of health promotion. However, taking a closer look at the nursing students’ PA behavior reveals that they already demonstrate comparably very high volumes of PA throughout their day [14, 15]. Against the background of the PA paradox positing that occupational PA does not entail similar health effects than leisure-time PA [16, 17], it appears valuable in the field of nursing to not only target levels of PA but in particular to strengthen competencies and resources for coping with physical demands at work and adopting a physically active lifestyle. Indeed, a previous study demonstrated that such competencies pertaining to healthy, active lifestyles are more strongly associated with work ability and psychophysical health than the mere volume of PA [15]. Against this background, PA promotion has to reconsider the mere focus on activity volume in this target group.

**Fig. 1:**
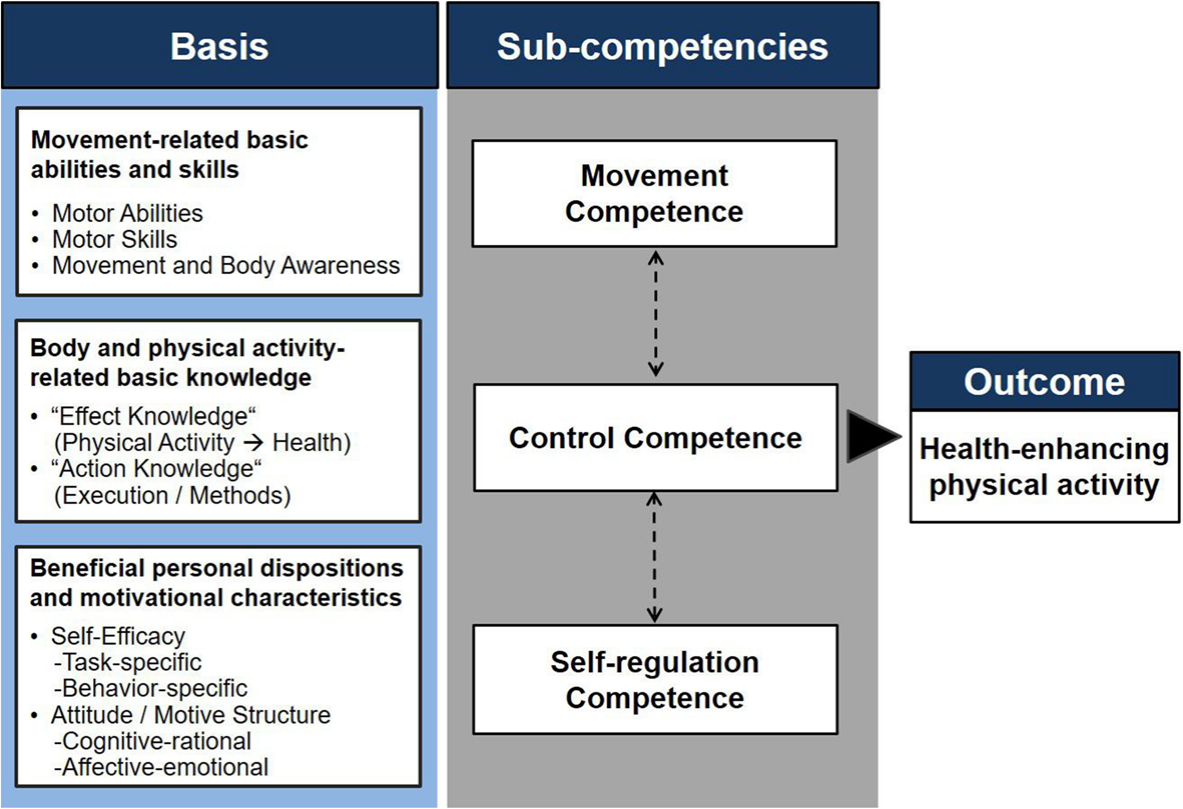
The physical activity-related health competence (PAHCO) model [18, 19]

From a theoretical perspective, the authors of the study have drawn on the physical activity-related health competence (PAHCO) model [18, 19]. The framework specifies three competencies which prepare individuals to meet physical demands of daily life and lead healthy, active lifestyles (see Fig. 1). *Movement competence* addresses the mastering of directly movement-related tasks and guarantees that individuals can tolerate challenging physical demands, perform activities in daily life, and participate in planned exercise. *Self-regulation competence* covers the motivational and volitional requirements by ensuring the regularity of taking up, maintaining, and enduring physical activities. Lastly, *control competence* does not unreflectively follow the slogan “the more, the better” but takes into account that activity modes and loads align with the idiosyncratic individual requirements to achieve positive effects on holistic health (i.e., physiological, social, psychological). These three major model components of the PAHCO model are, in turn, formed by the integration of basic abilities and skills (e.g., motives, endurance capacities, knowledge aspects, balance abilities, sensory integration, self-efficacy, strength abilities, body awareness, pacing strategies).

In accordance with these descriptions, the model gains high relevance for the target group of nursing students. Nursing students should possess a conditional base to manage the range of physical challenges associated with walking around, mobilizing and washing patients, or lifting objects (movement competence) [20]; from a psychological point of view, they have to persevere the pace throughout the working day for providing care to patients and be active in leisure time beyond the working schedules (self-regulation competence) [21]; they should also know the most suitable ergonomic techniques and be able to economically distribute their energetic resources throughout the entire day (control competence) [22].

PAHCO has been adopted as a curricular content for generalist vocational nursing training in Bavaria, Germany, in 2020 [23]. However, it cannot be assumed that PAHCO has yet comprehensively permeated the educational routines at nursing schools in Bavaria. Instead, key adjustments referred to the transition from specialized nursing programs (i.e., nursing, pediatric nursing, geriatric nursing) to generalist nursing programs. This prioritization may have counteracted endeavors to systematically integrate learning sessions and modules addressing students ‘ holistic coping with physical demands. Therefore, the educational landscape can benefit from explicit recommendations and materials that facilitate the adoption of PAHCO in vocational nursing training.

### Implementing physical activity promotion in nursing schools

When searching for adequate approaches eligible to foster PAHCO, the scientific literature on health promotion offers different solutions for setting-based PA promotion [24, 25]. Regardless of whether these approaches, for instance, more strongly emphasize the self-organization and self-responsibility (autopoiesis) of systems or the need to change the structure and environment itself (structural determinism), researchers cannot give universal recommendations in regard to the best effectiveness [26]. Instead, the selection of the most suitable approach depends on the balances of the corresponding strengths and weaknesses as well as the fit with the context and the values of stakeholders [24]. However, according to a well-established dichotomy, literature frequently distinguishes between top-down approaches, on the one hand, and bottom-up approaches, on the other [27, 28]. *Top-down approaches* can be characterized by experts systematically gathering information about the target group and deriving interventions based on the aggregation of available evidence of the literature. In this context, organizations are given the responsibility to incorporate the expert-based intervention into their setting, ideally for long-term impact. In contrast, *bottom-up approaches* emphasize the need that interventions must fit perfectly with the unique conditions of a setting. Accordingly, these types of interventions follow participatory ideas and consistently integrate relevant stakeholder during intervention development and implementation [29, 30]. Although there are sufficient theoretical arguments inducing researchers to favor one of these approaches, it is from an empirical perspective merely possible to clearly assign priority to one of two sides. In this regard, the present study aiming to strengthen PAHCO within the educational landscape of nursing in Bavaria, Germany, cultivates an open attitude toward PA promotion by comparing different interventions approaches along the spectrum of bottom-up and top-down solutions.

### Goals and hypotheses

The first goal of the present study is to gain insights regarding the best intervention approach for promoting PAHCO in nursing schools. The present study compares three different intervention groups (IG-1, IG-2, IG-3) with a control group (CG) that does not undergo any further intervention beyond regular school education and activities. Accordingly, the research question of this study is *Which is the most convenient intervention approach to foster PAHCO in vocational nursing training?* We hypothesize that, after controlling for baseline values, individuals in the three intervention groups show higher PAHCO values after the intervention (T2, T3) than their counterparts in the CG. We do not specify further hypotheses regarding the superiority of any intervention group but compare the intervention-induced effect size across the different study arms.

The second goal of the present study is to examine the implementation of different intervention approach in the nursing landscape. Therefore, a further research question of this study is *How are the different interventions implemented within nursing schools?* As we conduct qualitative analyses with multiple data sources, we do not test any specific hypotheses.

## Methods

The present study protocol is informed by the 2013 Standard Protocol Items—Recommendations for Interventional Trials (SPIRIT) guideline, suggesting 33 items for the comprehensive reporting of trials (see also Supplementary Table 1) [31]. Accounting for the specificities of the study design, the final primary publication adheres to the CONSORT 2010 Statement, including its extended version to cluster randomized trials [32]. The study has been approved by the ethics committee of the medical faculty at Friedrich-Alexander University Erlangen-Nürnberg (No. 22–429-S), and data collection at schools has been granted by the Bavarian State Ministry of Education (IV.7-BO9106/144/9).

### Study design and setting

**Fig. 2:**
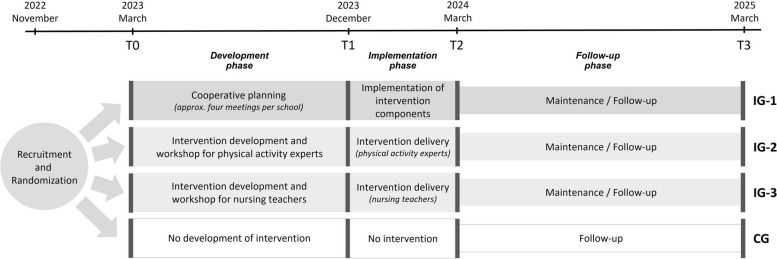
The study design including the four different study arms and measurement time points

**Fig. 3:**
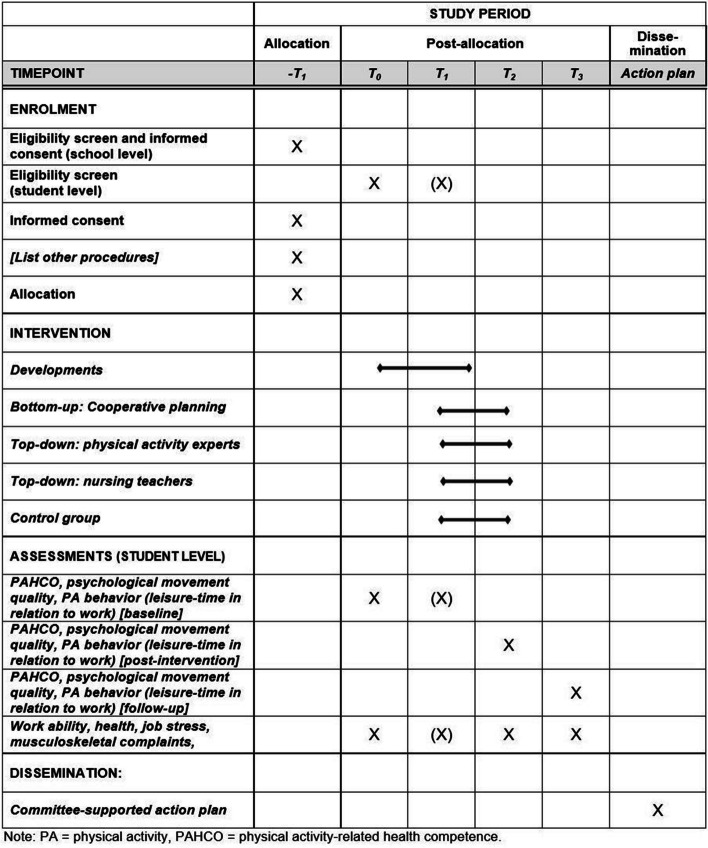
Schedule of enrolment, interventions, and assessments in accordance with the SPIRIT guidelines

We apply an effectiveness-implementation hybrid research design, combining simultaneous evaluation of intervention effectiveness and implementation [33]. The design contains a cluster randomized controlled trial (cRCT) with four parallel study arms to identify the most convenient approach for strengthening PAHCO. In summary, the study harbors four time points for measurements (Fig. 2): a baseline before intervention development (T0); after the intervention development but before the implementation (T1); directly after intervention implementation (T2); and 1 year after the implementation (follow-up; T3). An alternative depiction following the SPIRT schedule is presented in Fig. 3.

To acquire an overview of all nursing schools in Bavaria, we contacted the person responsible for nursing education within the Bavarian State Ministry of Education in the preparatory phase of the study. In September 2022, we obtained a list with a total of 185 schools that are targeted by the curriculum reform including PAHCO. To promote comparability, account for some potential confounders (through better homogeneity), and enable better causal inferences, we decided to reduce variance across schools by specifying further criteria for the inclusion into this trial (see eligibility in the next section). We randomly contacted 16 (see sample size calculation in chapter 2.6.1) of all 61 schools meeting these eligibility criteria and asked them for participation in this study. After a time period of 3 weeks without any response, the research team undertook reminder calls via telephone. For each school, that did not agree to participate in the study, we contacted a new school meeting the inclusion criteria (the sequence was also randomly generated a priori) and asked for participation. In this way, the research team recruited a total of 16 vocational schools in Bavaria, Germany, for the trial. After gaining written informed consent for study participation, we randomly assigned these 16 schools to four different study arms for testing different intervention approaches to strengthen nursing students’ PAHCO.

### Eligibility criteria

Due to the cluster design of the present study, we formulated eligibility criteria on the school and the student level. We only included *schools* (a) with a private or local sponsorship, (b) that regularly have two or three classes per year (according to the list of schools provided by the Bavarian State Ministry of Education), equaling between 33 and 64 students per year, and (c) whose school director provided informed written consent to participation by March 1, 2023, at the latest. Moreover, we only included *students* who (i) provided informed written consent to participate (and additionally their legal guardians, if aged under 18 years), respectively, and (ii) were part of the first year of vocational nursing training (within the school cohort starting in September 2022 or April 2023). Any schools and students not meeting all criteria were excluded from the study.

### Interventions

In IG-1, each school takes part in a *bottom-up* process to develop interventions tailored to the target group and school. In this case, the co-creation approach “cooperative planning” (CP) is adopted in which various relevant actors from research, policy, and practice are involved in an equal decision-making process for planning, developing and implementing interventions [34, 35]. CP accounts for the complexity of health promotion by jointly defining health-related goals through the cooperation of actors from science, practice, and policy [34, 36]. Health-oriented PA promotion in Germany has not only repeatedly used this specific method [37–39] but also identified vocational nursing training as an adequate sector for applying CP [40, 41]. A CP process comprises three successive phases: the preparation, planning, and implementation phase [34, 42, 43]. The *preparation phase* marks the starting point of a CP process and aims at getting to know the target group and the specific setting (i.e., in our case the nursing students and nursing schools). Furthermore, this stage includes the identification of relevant actors eligible to be involved in the subsequent planning phase (e.g., nursing students, nursing teaches, headmaster). The *planning phase* represents the main part of the CP process and encompasses the collaborative development of interventions and intervention components. Usually, four to six CP meetings take place at the respective nursing school over a period of 6 to 12 months. The planning phase commences with a “brainstorming” to accumulate ideas on potential interventions (e.g., to promote PA or PAHCO). Subsequently, the group discusses and prioritizes the generated ideas by weighting their importance and feasibility. The panel specifies and elaborates the tangible interventions, which finally result in a recorded action plan. This action plan contains a description of the goals and contents of the single interventions as well as information on the implementation steps (e.g., further requirements for the implementation, defined time frames and responsibilities). In the concluding *implementation phase*, the practitioners take the responsibility to implement the developed interventions according to the action plan, supported and advised by the researchers as required [34, 42, 43]. In the follow-up period, nursing schools are requested to implement the interactively defined intervention components under self-responsibility.

In line with a *top-down approach*, the schools of IG-2 and IG-3 receive an expert-based intervention. For the development of this intervention, the research team draws on intervention mapping [44, 45] as a processual framework providing six steps (see Supplementary Table 2) for the planning of theory-based and evidence-based programs: logic model of the problem (step 1), program outcomes and objectives (step 2), program design (step 3), program production (step 4), program implementation plan (step 5), evaluation plan (step 6). Consistency throughout the intervention development process was ensured by cultivating explicit links of the theoretical sub-components of PAHCO (i.e., movement competence, control competence, and self-regulation competence) with the objectives and content of the intervention (Table 1). As part of the intervention mapping process, we consulted independent experts external to the research team with in-depth knowledge in nursing care, health, and PA promotion. The entire intervention comprises 12 sessions of 90 min each and is described in detail in a delivery manual. An overview of the intervention sessions is given in Supplementary Table 3. The schools of IG-2 are sent external physical activity experts, who additionally undergo specific PAHCO training in a short online workshop and facilitate the program after making temporal and organizational arrangements (e.g., sports hall, equipment, timing) in direct interaction with the local nursing schools. The schools of IG-3 are facilitated by teachers from the vocational schools. The respective schools self-define eligible teachers for the delivery, who participate in a 1-day workshop on PAHCO and the expert-based program. Afterwards, these teachers are advised to integrate the sessions into the regular education schedule of their school. Except of the empowering nature of the PAHCO concept, the research does not implement strategies for improving adherence beyond the scheduled sessions in IG-2 and IG-3. If the schools are interested in using the expert-driven PAHCO intervention also in follow-up period, they themselves have to cover the financial and temporal resources for its maintenance.

**Table 1:** Overview of the intervention content

Explicit links to the sub-components of the PAHCO model	Performance objectives	Sample intervention contents [session number]
Movement competence during leisure time	Students adequately manage the immediate movement-related demands of leisure-time physical activity	• Prompt execution of different types of physical activity and exercise [1–12] • Information about the execution of different strengthening exercises and prompt their rehearsal [5–7]
Movement competence during work	Students manage the immediate movement-related demands of physical activity during nursing work	• Prompt execution of different types of physical activity and exercise [1–12] • Information about exercises that train muscles needed for movements during nursing work and prompt their practice [5, 7]
Control competence during leisure time	Students adequately direct their leisure-time physical activity towards maintaining or promoting health and well-being	• Provide information about effects of PA and exercise [2] • Prompt application of methods to determine and control exercise intensity [3, 4, 7]
Control competence during work	To the extent possible, students direct their physical activity during nursing work towards maintaining or promoting health and well-being	• Information about the execution of different lifting and carrying techniques and prompt their practice [6] • Prompt reflection of ways to maintain or promote one’s personal health and wellbeing during nursing work [12]
Self-regulation competence during leisure time	Students ensure the regularity of leisure-time physical activity	• Prompt development of personal SMART goals [9] • Planning personal physical activity at a particular time, on certain days of the week, and a specific place [11]

*PAHCO* Physical activity-related health competence

The schools of the CG do not receive any structured intervention content from external by following regular education processes and potential health promotion at the nursing schools. Although nursing schools themselves could basically take initiatives to align practices with the PAHCO concept as suggested within the state curriculum, current screenings did not uncover any specific recommendations and material for the implementation of PAHCO.

### Assignment to interventions and blinding

We perform randomization on the nursing school level. After 16 school directors provided consent for their nursing schools to participate in the study, we allocate these schools to the four study arms using a balanced randomization function in Microsoft Excel 2016 (Microsoft Corporation, Redmond, USA). The nursing students have an active participation role and are informed about the intervention and control groups, which undermines potential blinding on the recipient level. Given the bottom-up character of IG-1, researchers as well as school directors, teachers, and nursing students become even integral part of the intervention development through co-creation. Furthermore, the deliverers in IG- 2 and IG-3 (i.e., external PA experts, nursing teachers) are aware of the respective intervention and cannot be blinded. For pragmatic reasons, blinding can also not be ensured for the assistants instructing the paper–pencil assessments. The research team conducts the statistical analyses internally.

### Data collection and management

In line with the effectiveness-implementation hybrid research design [33], we collect data to evaluate the effectiveness of the study arms, on the one hand, and aspects of implementation, on the other. For pragmatic reasons, the schools can decide whether they prefer to conduct the data collection analogously (via paper–pencil procedures) or digitally (via online survey; SoSci Survey GmbH, Munich, Germany). At the beginning of each assessment, all participants are asked standardized, (person-)invariant questions to generate a central pseudonym and detach from handling tangible names (ensuring confidentiality). Data collection for nursing students is organized via self-defined contact persons at the participating schools (i.e., distribution of questionnaires and links, scheduling of filling slots). An overview of all assessments including their time point is given in Table 2.

**Table 2:** Overview of the quantitative and qualitative assessments

Construct	Assessment and reference	Time point			
		T0	T1	T2	T3
Participant characteristics	Self-report [gender, age, height, weight, year of education, highest educational degree, specialization of nursing education, school affiliation]	X			
**Primary outcomes**					
Physical activity-related health competence [PAHCO]	PAHCO questionnaire	X	X	X	X
Movement quality	Physical Activity Enjoyment Scale [PACES] (specified for two different contexts, i.e., leisure time and work)	X	X	X	X
Leisure-time physical activity	BSA Questionnaire	X	X	X	X
Occupational physical activity	The occupational sitting and physical activity questionnaire [OSPAQ]	X	X	X	X
**Secondary outcomes**					
General health status	Single item [item #1] of the SF-12 Questionnaire	X	X	X	X
Work ability	Work Ability Index, short form [WAI-r]	X		X	X
Musculoskeletal complaints	Nordic Musculoskeletal Questionnaire [NMQ]	X		X	X
Subjective job stress	Self-developed questionnaire with 30 items [validation with T0 data]	X		X	X
**Processual variables**					
Readiness for change at nursing schools	Wandersman Center’s Readiness Thinking Tool [RTT]	X			
Implementation of interventions	Immediate: session protocols, self-developed questionnaire [informed by the subcategories “training providers” and “delivery of treatment”]		X	X	
	Long term: self-developed questionnaire		X	X	
Determinants of intervention implementation	Interviews				X

#### Effectiveness data

##### Basic participant characteristics

We acquire the following basic participant data via self-report: gender, age, height, weight, year of vocational nursing training, highest educational degree, specialization of vocational nursing training (i.e., generalist nurse, pediatric nurse, geriatric nurse), and school affiliation.

##### Primary outcomes

We employ the 42-item PAHCO questionnaire [46, 47]. The questionnaire has already been specifically validated for the target group of nursing students [46] and, based on analyses of the factorial structure with parallel loadings, allows the aggregation of separate scores for movement competence (21 items across five scales), control competence (13 items across three scales), and self-regulation competence (14 items across four scales).

Movement quality—in a psychological/affective sense—goes beyond PAHCO by not covering how individuals are *able* to align PAs toward psychological health but rather how they actually *perceive* enjoyment. Movement quality is operationalized via the German version of the Physical Activity Enjoyment Scale (PACES) for adults [48]. The instrument comprises 16 items to be answered on a 5-point Likert scale; previous validations [48] with adolescents and young adults have focused on age groups similar than in the present study. We have generated two versions of this questionnaire, differentiating between perceived enjoyment of activities in leisure time and at work.

We strive for a comprehensive assessment of PA behavior. The BSA Questionnaire has been validated in German language and measures individual’s leisure-time and sport activity behavior of the past 4 weeks [49]. In addition, we apply the Occupational Sitting and Physical Activity Questionnaire (OSPAQ) with its four activity sub-categories sitting, standing, walking, and performing heavy labor at the workplace [50]. Taking into account the discussions of a PA paradox suggesting that PA in the occupational context may not provide the same health benefits as demonstrated for leisure-time PA [16, 17, 51, 52], we deliberately prioritize leisure-time PA in its relation to occupational PA in our analyses. We build an index expressing the portion of leisure-time/sport activity in relation to overall PA: PA_BSA_/(PA_BSA_ + PA_OSPAQ_).

##### Secondary outcomes

We draw on a multidimensional strategy to assess stress-related outcomes at work. More specifically, we measure nursing students’ work ability using the reduced version of the Work Ability Index (WAI-r) in German language [53]. The WAI-r contains five dimensions with eight items. The Nordic Musculoskeletal Questionnaire (NMQ), which has recently been translated into German [54], systematically asks participants about their complaints across ten different body regions. We register students’ general health status with a single item of the SF-12 Questionnaire (item #1) [55]: “How would you describe your general state of health?” [Original Question in German: “Wie würden Sie Ihren Gesundheitszustand im Allgemeinen beschreiben?”]. The question has to be answered on a five-point Likert scale and has already been used in a PAHCO study in exactly this variant [56]. Finally, we apply a 30-item questionnaire recording the subjective job stress during vocational nursing training. This questionnaire has been conceptualized within a previous project using three focus groups, and psychometric aspects are explored with the baseline data (T0) of this project.

#### Implementation data

Prior to the intervention, the directors and teachers from the schools in all study arms self-evaluate the readiness of the school to undergo any change for the implementation of PAHCO as a curriculum content in vocational nursing training. We employ the Wandersman Center’s [57] Readiness Thinking Tool (RTT) which is based on the *R* = MC^2^ framework by Scaccia et al. [58]. This tool contains 19 items to assess the components “motivation,” “innovation-specific capacity,” and “general capacity.” As we could not find a German version, we translate the RTT using forward and back translation with monolingual tests [59]. A native German speaker undertakes the forward translation, and the back translation is done by a native English speaker. Subsequently, two researchers validate and discuss both versions to refine the RTT in German language. Following the Readiness Diagnostic Scale [60], we use a seven-point Likert scale instead of the original four-point Likert scale to allow for a more precise assessment. Moreover, we add an item asking for activities promoting PA and health that have already taken place in the participating institutions.

During the interventions, in the CP sessions of IG-1, employees of the research team prepare structured protocols to record the date and duration of meetings and the participation of stakeholders as well as key content and decisions during the process. The developed PA interventions and intervention components are captured via action plans at the end of the planning phase.

In IG-2 and IG-3, the external PA experts and nursing teachers complete a standardized protocol after each intervention session to document intervention implementation, including the following information: the number of participants, unusual incidents, potential divergences from the intervention manual, a self-reflection of teaching performance as well as short, day-specific evaluations of student behaviors, perceived sovereignty, and an overall evaluation. After the final session, they additionally answer a five-item survey on treatment fidelity (informed by the important subcategories “training providers” and “delivery of treatment” [61, 62]).

After the implementation and follow-up phase, we conduct retrospective interviews with relevant actors of all 16 schools to capture central determinants and influencing factors for the implementation of the interventions. An exemplary question is “What has contributed to the intervention(s) being implemented/not implemented?”.

### Data analyses

#### Effectiveness data

##### Primary analyses with sample size calculation

PAHCO (including its three model components), the leisure-time/sport versus occupation PA index, and psychological movement quality serve as primary outcomes, both at T2 (post-implementation) and at T3 (follow-up). To illuminate the differential change evoked by the interventions, we run linear mixed models separately adopting a short-term (T2) and medium-term (T3) perspective. Setting-specific readiness for change (via RTT) is considered to be treated as a covariate on the cluster level for minimizing the potential bias that intervention effects may be referred to significant institutional differences in this variable across the study arms. In case of rejected equality of variances across time (as indicated by Mauchly’s Test for Sphericity), we apply corrections or models with robust assumptions. Main attention is paid to group*time interactions, expressing differential developments of indicator measurements over time. We include all participants into the analyses who initially met the inclusion criteria and participated in the baseline assessment (intention-to-treat assumption). Accordingly, all potential missing data are handled using imputation procedures [63]. We conduct all calculations in the open-source software R (version 4.1.3 or higher). All quantitative questionnaire data are stored and processed in SPSS (IBM, Armonk, USA) after cultivating double checks of data entry with at least one other assistant.

The present trial involves four study arms and four measurement time points (T0, T1, T2, T3). Informed by previous experiences in the context of PAHCO, we anticipate longitudinal construct stability of *r*_t_ = 0.50 (autocorrelation). Calculations in G*Power (v3.1) [64] with an assumed a power (1 − *β*) of 0.80 revealed that we require a sample size of *n* = 200 for registering at least a small effect (*d* ≥ 0.20, *f* ≥ 0.10) in comparison to the CG [65]. However, given that nursing students are nested within schools and that the settings may assign different importance to health and PA promotion, we account for clustering (ICC = 0.02) by considering a design effect of DE = 1.78 (*n*_cor_ = 356). In addition, we consider a substantial rate of non-consent to participation (20%) in the first step and longitudinal dropout (30%) in the second step, resulting in *n*_final_ = 636. On average, Bavarian nursing schools have 20 students per class and, in turn, two classes per cohort (corresponding to 40 annual students per year). Given these sample size calculations, we have to recruit a total of 16 schools for the present trial (resulting in an overall potential of 640 nursing students).

##### Secondary analyses

We explore post-intervention differences between the study arms also with the secondary outcomes (e.g., general health, work ability, musculoskeletal complaints, job stress). The longitudinal character of this study allows to perform additional analyses relevant for PA and health. For instance, the research team is interested in examining the reciprocal relationship between PAHCO and PA by conducting cross-lagged panel analyses. Based on initial path analyses underlining the relevance of PAHCO for students’ health [15], we could investigate the mediating role of PAHCO in the association between nursing-related job stress and work ability. The integrated T1 measurement also allows to investigate whether potential improvements in PAHCO at T2 might be affected by an empowerment of the schools already during the development phase. Finally, it would be worth retrospectively linking the degree of implementation to differential intervention effectiveness across nursing schools.

#### Implementation data

We descriptively analyze the readiness of all schools using the data from RTT. Average readiness scores are calculated across the components “motivation,” “innovation-specific capacity,” and “general capacity,” which range on a scale from 1 (indicating low readiness) to 7 (indicating high readiness). Descriptive analyses of the structured protocol deliver information about the implementation of interventions in IG-1. For example, we report the number of meetings and characteristics of involved stakeholders. The action protocols are analyzed descriptively (e.g., numbers of newly developed PA interventions) and qualitatively (e.g., comparison of the interventions’ characteristics across all schools of IG-1). We descriptively analyze data from standardized protocols, reporting the number of participants, characteristics of the sessions, self-rated teaching performance, student behavior, perceived sovereignty, and an overall evaluation of the sessions to examine the implementation of interventions in IG-2 and IG-3. Similarly, we descriptively explore data from the treatment fidelity survey. The software SPSS (IBM, Armonk, USA) and Microsoft Excel 2016 (Microsoft Corporation, Redmond, USA) serve to conduct the analyses. The interviews on factors influencing the intervention implementation with relevant actors of all 16 schools are audio-recorded and transcribed verbatim. To ensure anonymity, we replace personal names with working positions and school as well as city names with pseudonyms. Subsequently, we submit the transcripts to qualitative content analysis [66]. We apply MAXQDA (VERBI Software, Berlin) for the transcription, data coding, and analyses of the interviews. Finally, the quantitative and qualitative data are triangulated at the interpretation stage [67] to describe the implementation of interventions in detail.

### Dissemination plans

The findings of this study combining effectiveness and implementation aspects dictate the recommendations with respect to the dissemination of PAHCO in vocational nursing training in Bavaria. The “practical planning for implementation and scale-up” (PRACTIS) guide provides comprehensive guidance for translating evidence-based PA interventions into practice [68]. It describes an iterative four-step process to (1) characterize the parameters of the implementation setting, (2) identify and engage key stakeholders across multiple levels within the delivery system(s), (3) identify contextual barriers and facilitators to implementation, and (4) address potential barriers to effective implementation [68]. In addition to researchers, stakeholders should be involved in all four steps. For this purpose, a steering committee is established, consisting of various actors from research, policy, and practice. Finally, we will develop recommendations for action that describe how to implement the most effective and sustainable intervention (i.e., the activities of IG-1, IG-2, or IG-3), aiming to implement the intervention across Bavaria and reach more nursing students. We publish the study results in peer-reviewed journals and present the findings at scientific conferences on the national and international scale.

## Discussion

The present study aims to test three different intervention approaches to enhance PAHCO among nursing students. The design of these interventions is explicitly aligned with the bottom-up approach (IG-1) for health promotion, on the one hand, and to the top-down approach (IG-2 and IG-3), on the other. In this respect, the current endeavor holds an open attitude toward beneficial interventions for the target group, which has to cope with physical demands and use PA for a healthy lifestyle throughout their professional career. Differentiating between short-term and medium-term effects of the intervention, we are interested in deriving the most appropriate approach for implementing PAHCO in nursing schools. In this regard, the study findings can substantially inform a subsequent dissemination of PAHCO on a larger Bavarian (Germany) scale. The insights of this study may contribute to better implementation and transfer of PAHCO into practice, to higher quality in vocational nursing training, and, in the long term, to a more pronounced health promotion for the nursing staff.

The present study bases on a comprehensive recruitment of nursing schools in Bavaria. While the provision of consent appears realistic for the intended number of schools, a decisive factor for the achievement of the calculated sample size could be the number of classes for each cohort. Although the Bavarian State Ministry of Education has delivered a list of schools engaging at least two classes, it cannot be excluded that single schools may only have one class in the respective intervention year (e.g., due to missing student enrolment) or where single classes are not participating for any reasons (e.g., organizational problems). Therefore, the clustered recruitment represents a crucial factor within this study. Nevertheless, the results of this hybrid study design could not only serve to arrange effective and implementable initiatives in curriculum-embedded vocational nursing training but also to better understand paradigmatic approaches (top-down versus bottom-up) in the context of setting-based physical activity promotion.

## Trial status

This trial has been registered online (first registration on March 14, 2023; published and latest update on April 18, 2023) at ClinicalTrials.gov NCT05817396 (https://clinicaltrials.gov/study/NCT05817396), and this article presents the first protocol version. Baseline data collection started on March 1, 2023 (with the beginning of T0), and is planned to be ended on December 22, 2023 (with the end of T1), at the latest. The data collection of the follow-up period will end April 30, 2025. As this study protocol reports already the content of the developed interventions as a basis for the following implementation phase (see the entire “Interventions” section), it was not possible to submit this protocol at an earlier time point of this study (Fig. 2).

### Supplementary Information

Supplementary Material 1.

Supplementary Material 2.

## Data Availability

The datasets analyzed during the current study and statistical code are available from the corresponding author on reasonable request, as is the full protocol.
