# Supplementary material for: Implementation and dissemination of physical activity-related health competence in vocational nursing training: study protocol for a cluster-randomized controlled intervention trial
Source: Trials. 2024 May 15;25:322. doi: 10.1186/s13063-024-08153-2 (PMC11094863; doi:10.1186/s13063-024-08153-2)
Supplement: Supplementary file 2 — Supplementary Material 2. [file 13063_2024_8153_MOESM2_ESM.docx]

| In step 1, the intervention planning team establishes a planning group including relevant stakeholders that will participate in the intervention development process. Furthermore, a needs assessment is conducted to create a logic model of the problem, the intervention context is described and program goals are stated. Step 2 includes the specification of program outcomes and objectives as well as the construction of matrices of change of objectives that results in the creation of a logic model of change. Program design (step 3) includes the generation of program themes, components as well as the program scope and its sequence. Theory- and evidence-based methods to attain desired objectives are chosen and ways to implement them defined. In step 4, the program structure is refined and program materials planned, drafted, pretested, refined and produced. The program implementation plan (Step 5) is developed by identifying potential program users, stating outcomes and objectives for program use, constructing matrices of change objectives for program use, and designing implementation interventions. To develop an evaluation plan, steps 6 includes writing evaluation questions, developing indicators and measures for assessment, specifying the evaluation design and completing the evaluation plan. |
| --- |

**Table 2.** The six steps of intervention mapping as a framework for the expert-based interventions.

**Table 3.** The different sessions of the interventions and their specific goals.

| **Session Number** | **Subject** | **Session Goals** |
| --- | --- | --- |
| 1 | Movement Games I – Mood Management | 1. The students are familiar with various forms of physical movement. 2. The students are aware of the effects of physical activity on mood. 3. The students describe the effects of physical activity on their mood and evaluate physical activity as something positive. |
| 2 | Movement Games II – Effects of Physical Activity | 1. The students are familiar with various forms of physical exercise that serve to improve their strength, endurance, and coordination. 2. The students are aware of the positive effects of regular physical activity. 3. The students differentiate the specificities of physical activity in a professional context. |
| 3 | Endurance I – Strain & Exertion | 1. The students are familiar with forms of exercise that serve to improve their endurance. 2. The students apply different methods to assess exertion. 3. The students are aware of the physiological effects of endurance training. |
| 4 | Endurance II – Exertion Control | 1. The students are familiar with various forms of physical exercise that serve to improve their endurance. 2. The students know the fundamentals of endurance training and outline different methods of endurance training. 3. The students control the intensity of endurance training based on their pulse and perceived exertion. 4. The students identify endurance training as a means to counteract the demands of the nursing profession. |
| 5 | Strength I – Movement Patterns | 1. The students are familiar with different exercises that serve to improve their strength and flexibility. 2. The students know the basics of strength training and perform key features of selected strength and mobilization exercises. 3. The students differentiate the workload profile of a caregiver in the context of fundamental movement patterns. |
| 6 | Strength II – Back Health | 1. The students are familiar with different exercises that serve to improve their strength and body awareness. 2. The students know the physiological and anatomical fundamentals of the spine. 3. The students are familiar with key features of various lifting and carrying techniques and apply these techniques while considering these features. |
| 7 | Strength III – Exertion Control | 1. The students perform self-selected resistance exercises for the maintenance and promotion of caregivers’ health, aimed at improving their strength. 2. The students knowthe basics of resistance training. 3. The students control the intensity of their workload in resistance training based on their perceived excertion. 4. The students identify resistance training as a means to counteract the physical demands of the nursing profession. |
| 8 | Activation & Relaxation – Affect Regulation | 1. The students are familiar with forms of exercise and relaxation techniques aimed at intentionally altering their state of tension. 2. The students are aware of the various effects of physical activity on their well-being. |
| 9 | Coordination I – Motives, Preferences & Goals | 1. The students are familiar with various exercises that serve to improve their coordinative abilities. 2. The students reflect on their physical activity behavior using a physical activity diary. 3. The students analyze their personal motives for physical activity and movement preferences. 4. The students are aware of the principles of goal setting and define their own goals for physical activity. |
| 10 | Coordination II –Movement Opportunities | 1. The students are familiar with various exercises that serve to improve their coordinative abilities and body awareness. 2. The students know the fundamentals of action planning with a focus on identifying movement opportunities. |
| 11 | Coordination III – Action Planning & Barrier Management | 1. The students are familiar with various exercises that serve to improve their coordinative abilities and body awareness. 2. The students know the fundamentals of action planning and create their own action plan considering personal motives, goals, and barriers. |
| 12 | Movement Games III – Reflection on Past and Future Movement Behavior | 1. The students are familiar with various forms of exercise that serve to improve their motor skills. 2. The students reflect on the significance of physical activity in the context of their biography. 3. The students identify physical activity as a means to counteract the demands of the nursing profession and promote their health. |
